# Medical Progress

**Published:** 1877-10

**Authors:** 


*Medical Progress.*—Considerable discussion has been going on in Great Britiain, in the past few months, growing out of an offer on the part of a distinguished homœopathic physician, and a representative man of that school of medicine, to unite the opposing "pathies," or at least bring about a reconciliation between them. The doctrines of Hahneman are scarcely held now by any save the most exclusive, and we had almost said, ignorant of those practicing under that name, and certainly the day of heroic dosing on the part of the allopaths seems over, a result they are not entirely unwilling to credit the other parties with. Absolutism disappears and the field of toleration widens as real scientific knowledge is gained, and we know as dentists, that homœopathy has furnished us with most careful hints in the management of diseases of the teeth. It is not probable that such a union as is proposed will be effected at present at least, but the fact that it is even suggested is an admission of a great advance in medical toleratiou.

---

In the *Monthly Summary* will be found a brief abstract from a work by a prominent female physician, of New York City, on the question of "rest during menstruation." As so many females from foreign countries are entering upon the study of dentistry, the conclusions arrived at are important. The book in question is a thoroughly exhaustive and scientific treatise on a subject the writer has had special advantages for investigating. The work may be worth the attention of those who are interested in entering upon a profession so severely taxing the energies, and involving so much standing upon the feet.

